# Effectiveness of raw bacteriocin produced from lactic acid bacteria on biofilm of methicillin-resistant *Staphylococcus aureus*

**DOI:** 10.14202/vetworld.2023.491-499

**Published:** 2023-03-21

**Authors:** Hanaa Khaleel Ibraheim, Khadeeja S. Madhi, Gaida K. Baqer, Hasanain A. J. Gharban

**Affiliations:** 1 Department of Microbiology, College of Veterinary Medicine, University of Basrah, Basrah, Iraq; 2 Department of Microbiology, College of Medicine, University of Basrah, Basrah, Iraq; 3 Department of Internal and Preventive Veterinary Medicine, College of Veterinary Medicine, University of Wasit, Wasit, Iraq

**Keywords:** *16S rRNA* gene, biofilm formation assay, *mecA* gene, polymerase chain reaction, subclinical mastitis

## Abstract

**Background and Aim:**

Probiotics are proven beneficial to health since they enhance immunity against dangerous pathogens and increase resistance to illness. Bacteriocin produced by lactic acid bacteria (LAB), demonstrates a broad inhibitory spectrum and therapeutic potential. This study aimed to isolate LAB-producing bacteriocin and investigate the effect of crude bacteriocin on biofilm from methicillin-resistant *Staphylococcus aureus* (MRSA).

**Materials and Methods:**

This study used randomly collected 80 white soft local cheeses (40 each from cows and sheep) from different supermarkets in Basrah Province. The obtained samples were cultured and the bacterial suspension of *S. aureus* was prepared at 1.5 × 10^8^ cells/mL. The crude bacteriocin extracted from LAB was obtained, and the tube was dried and inverted to detect the biofilm loss at the bottom.

**Results:**

There were 67 (83.75%) LAB isolates. Among 40 milk samples collected directly and indirectly, there were 36 (83.33%). *Staphylococcus aureus* isolates based on conventional bacteriological analysis and biochemical tests. Molecular testing was conducted to identify LAB and MRSA. Depending on genotypic results, the effect of white soft local cheese (cows and sheep) and the amplification results of the *16S rRNA* gene were detected in 46 LAB isolates from white soft local cheese from cows and sheep. Based on the molecular identification of the *mecA*, results on *Staphylococcus* determined that only 2 of 36 isolates of *S. aureus* carried the *mecA*. Moreover, there were 26 (86.66%) isolates (MRSA) from samples of raw milk from local markets and subclinical mastitis in cows. The ability of LAB isolates was tested. The effects of bacteriocin production on preventing biofilm growth and formation were investigated. Results demonstrated that bacteriocin has high activity. Microtiter plates applied to investigate the ability of *S. aureus* to produce biofilms revealed that all isolates were either weak or moderate biofilm producers, with neither non-biofilm nor strong biofilm producers found among the tested isolates.

**Conclusion:**

Lactic acid bacteria demonstrate a high ability to produce bacteriocin. Crude bacteriocin from LAB has a restrictive effect on biofilms produced by MRSA; thus, it can be used to reduce the pathogenicity of this bacterium.

## Introduction

Lactic acid bacteria (LAB) have a long history of use as probiotic bacteria in various environments, including the gastrointestinal tract, oral cavities, and vaginal tracts of humans and animals and in fermented food, silages, and composts [1, 2]. Lactic acid bacteria involve a group of Gram-positive, cocci or rod-shaped, anaerobic, and non-spore-forming bacteria that produce lactic acid as a main byproduct during carbohydrate fermentation [3]. These bacteria are important to health, as they prevent the development of pathogenic and dangerous microorganisms, enhance immune function, and raise resistance to illness [3]. As a source of vitamins and minerals, milk and its derivatives, such as yogurt, cheese, and dough, are crucial in the diets of several millions of people worldwide. Bacteriocin is an antibacterial peptide produced by LAB and used as a food preservative for controlling pathogen growth due to its broad inhibitory spectrum and therapeutic potential [4]. The primary structure of bacterial antibiotics determines how they work. Others can infiltrate the cytoplasm and affect gene expression and protein synthesis by penetrating the cytoplasm and releasing substances required for sensitive bacteria (cell lysis) [5]. Lactic acid bacteria bacteriocins are typically divided into three classes: Class I, which includes lantibiotics; Class II, which includes heat-stable unmodified bacteriocins; and Class III, which includes bigger heat-labile bacteriocins. Classes IIa-IIc are subsets of Class II. Class IIa bacteriocins are heat-stable, tiny (<10 kDa), unmodified peptides of 37–48 amino acids, which can prevent the growth of several food-spoiled and pathogenic bacteria, including *Listeria monocytogenes*, *Bacillus cereus*, *Clostridium perfringens*, *Staphylococcus aureus*, and *Escherichia coli* [6, 7]. They are the most investigated with respect to production and structure-function relationship and are considered one of the most interesting and potential groups of antimicrobial peptides for use in food preservatives [8]. Bacteriocins can inhibit the growth of closely related species (narrow spectrum) and species from different genera (broad spectrum). Their potential use as weapons against human, animal, and plant infections, as well as food spoilage microbes, has piqued scientists’ interest in recent years [9]. Bacteriocins have many advantages over conventional antibiotics, including the fact that they are directly gene encoded, allowing for bioengineering to improve their productivity or pathogen specificity [10, 11].

*Staphylococcus aureus*, one of the most significant facultative Gram-positive pathogens in human and veterinary medicine, causes various nosocomial diseases [12]. Due to its resistance to most antibiotics, including β-lactams, macrolides, and aminoglycosides, methicillin-resistant *Staphylococcus aureus* (MRSA) causes infections that are difficult to cure [13]. The disease known as bovine mastitis presents a significant health hazard to consumers and is the one that costs the dairy sector the most money globally [14]. *Staphylococcus aureus* is one of the most common bacteria found in intramammary infections in dairy cows, and the infections are typically subclinical and challenging to treat [15]. Enterotoxigenic *S. aureus* strains contaminate raw milk and dairy products. *Staphylococcus aureus* is a well-known main source of foodborne illnesses. Some of these strains could put humans at risk for food poisoning [16].

A biofilm is known as an extracellular polymeric matrix that protects a bacterial colony growing on biotic or abiotic surfaces [17]. The usual life cycle of *S. aureus* in the environment is thought to include biofilm development [18]. This barrier protects the bacterial community from the human immune system, adverse environmental conditions, and antibiotic assaults [19].

This study aimed to isolate LAB-producing bacteriocin and investigate the effect of crude bacteriocin on biofilm from MRSA.

## Materials and Methods

### Ethical approval

The present study was approved by and performed under the Scientific Committee of the College of Veterinary Medicine and College of Medicine, University of Basrah (Basrah, Iraq).

### Study period and location

The current study was conducted from April to July 2022 at the Colleges of Veterinary Medicine in the University of Basra and the University of Wasit.

### Sample collection

This study used 80 white soft local cheese samples randomly collected from different supermarkets in Basrah Province (40 each from cows and sheep). All samples were placed in sterile containers, kept in an ice box, and taken to the laboratory for analysis following an investigation. Directly and indirectly, 40 milk samples were collected, comprising 20 cow samples and 20 raw milk samples from local markets. The samples were taken from cows and tested for subclinical mastitis using the California mastitis test (CMT) [20].

### Identification and isolation of LAB

The obtained sample of white soft local cheese weighing 1 g was dissolved in 0.1% peptone water (9 mL), streaked over De Man, Rogosa, and Sharpe agar, and incubated for 2 days at 37°C in an anaerobic environment [21]. Gram-staining and primary cultures (white and cream colonies) were examined using colony morphology. Suspicious colonies underwent MRSA subculture and catalase biochemical testing.

### Isolation and characterization of *S. aureus*

All samples of indirect raw milk and milk that tested positive for CMT were analyzed bacteriologically by inoculation on mannitol salt agar and overnight incubation at 37°C under aerobic conditions. The colony shape and Gram-staining of primary cultures were examined. Using catalase, oxidase, and coagulase assays, the suspicious colonies were subcultured on mannitol salt agar [22].

### Polymerase chain reaction (PCR) confirmation of LAB isolates

At first, a DNA kit (Geneaid, USA) was used to extract the genomic DNA of all suspected isolates in accordance with the manufacturer’s instructions. The PCR MasterMix tubes were prepared for amplification gene primers were used for LAB bacteriocin creation (Tables-1 and 2) [16]. According to the gene-specific program (Table-3), the PCR tubes were transferred to a thermal cycler for an amplification reaction [23]. PCR products were electrophoresed on 2% agarose gel and visualized by ultraviolet (UV) light transilluminator.

**Table-1:** Primer sequence used in PCR detection of LAB.

Genes	Sequence	Amplicon Size	Reference
16S rRNA	F 5’- GCGGCGTGCCTAATACATGC -3’	700 bp	[16]
	R 5’- ATCTACGCATTTCACCGCTAC -3’		

PCR=Polymerase chain reaction, LAB=Lactic acid bacteria

**Table-2:** The PCR master mix preparation.

Material	Size	Reference
DNA template	5 µL	[16]
Master mix	12.5 µL	
Primer forward	1 µL	
Primer reverse	1 µL	
Nuclease free water	5.5 µL	
Total	25 µL	

PCR=Polymerase chain reaction

**Table-3:** Polymerase chain reaction conditions for 16S rRNA.

Stage	Step	Temperature (°C)	Time (min.)	No. of cycle
First	Denaturation	95	5	1
Second	Denaturation	95	1	30
	Annealing	42	1	
	Extension	72	1	
Third	Extension	72	10	1

### Extraction of crude bacteriocin

Crude bacteriocin from LAB isolated strain was grown in MRS broth seeded with 5% inocula of overnight culture and maintained aerobically for 24–48 h at 37°C. The entire broth was centrifuged at 6000× *g* for 15 min to obtain the supernatant. The supernatant was adjusted to pH 7.0, filtered, purified with 70% ammonium sulfate at 4°C, and recentrifuged at 6000× *g* for 15 min to precipitate the protein. The pellet was suspended in phosphate-buffered saline (PBS; 50 mL) to get crude bacteriocin [24].

### Genotypic detection of MRSA

Detection of the *mecA* and the primer sequences allowed for the molecular validation of MRSA isolates [4, 5]. With some modifications in the reaction conditions, PCR was conducted using the program described previously by Zhang *et al*. [25] (Tables-4, 5, and 6).

**Table-4:** Sequence and source of the gene primers for *mecA* Gene amplification.

Gene	Sequence	Amplicon size	Reference
*mecA*	F 5×-GTGAAGATATACCAAGTGATT-3’	147 bp	[25]
	R 5×-ATGCGCTATAGATTGAAAGGAT-3’		

**Table 5:** Polymerase chain reaction master mix for detection *S. aureus* MRSA isolates.

Material	Size
Master mix	12.5 μL
DNA template	2.5 μL
Primer forward	1.5 μL
Primer reverse	1.5 μL
Nuclease free water	7. μL
Total	25 μL

MRSA=Methicillin-resistant *Staphylococcus aureus,* *S. aureus=Staphylococcus aureus*

**Table-6:** Polymerase chain reaction amplification programs for *mecA* gene

Stage	Step	Temperature (°C)	Time (min.)	No. of cycle
First	Denaturation	95	5 L	1
Second	Denaturation	95	1 L	30
	Annealing	42	1	
	Extension	72	1	
Third	Extension	72	10	1

### *Staphylococcus aureus* sensitivity test to methicillin

The method used to detect MRSA was the MRSA resistance disk diffusion test. The culture was incubated on a Muller-Hinton agar plate for 18–24 h from 35°C to 36°C [26].

### Biofilm formation assay

The assay was conducted using the technique described previously [27]. Each strain was grown in tryptic soy broth (TSB). A microtiter plate (Costar, China) with three duplicate wells was used for each MRSA isolate after the culture had been diluted 1:100 in TSB supplemented with 1% glucose. The negative control wells comprised uncultured water (200 L; TSB supplemented with 1% glucose). Before being preserved with methanol (150 L) for 20 min, each well was cleaned thrice with sterile PBS (300 L; pH 7.2). The adherent biofilm layer in each well was colored for 15 min with 2% crystal violet (150 mL) after the plate was allowed to dry inverted for an additional day at room temperature. Then, 95% ethanol (150 mL) was added to each well after the microtiter plate had been rinsed thrice with PBS and dried at room temperature. The wells were read for 30 min. At 570 nm, the optical density (OD) was measured using the microplate reader (Biotek, USA) computations for the OD cutoff [28].

### Effect of crude bacteriocin on biofilm formation of *S. aureus*

Four LAB isolates were selected as the best isolates for bacteriocin production. The bacterial suspension of *S. aureus* was prepared after culture on brain-heart infusion broth at 37°C for 24 h. Then, a 1.5 mL bacterial suspension of *S. aureus* at 1.5 × 10^8^ cells/mL concentration with crude bacteriocin (1.5 mL) extracted from LAB prepared previously was obtained and incubated at 37°C for 24 h. The contents of the medium were poured out and washed with PBS. The tube was dried and stained with crystal violet at 0.1% concentration for 10 min, after which the dye was removed from the tube and washed with deionized water. Finally, the tube was dried and inverted to watch biofilm loss at the bottom.

### Statistical analysis

Fisher’s exact test, Pearson’s χ^2^ test, and Yates correction statistical package for the social sciences version 11 (IBM Corp., NY, USA) were performed to detect significant differences at p < 0.05.

## Results

### Bacteriological analysis and LAB identification

After employing MRS medium to isolate white soft local cheese from sheep and cows, white and cream colonies were found. Lactic acid bacteria isolates were identified by Gram-staining and catalase test. Sixty-seven of 80 (83.85%) isolates from LAB samples included 35 of 40 (87.5%) isolates of white soft local cheese from cows and 32 of 40 (80%) isolates of white soft local cheese from sheep. The isolates were identified as LAB characterized as Gram-positive, cocci, and rods, but most isolates were characterized as cocci, negative-catalase, and anaerobic.

### Detection of subclinical mastitis

California mastitis test results showed that 16 of 20 (80%) samples of direct milk were CMT positive, with weakly positive samples (21.25%), trace positive samples (53.25%), distinguishable positive samples (31.85%), and highly positive samples (63.75%; Table-7).

**Table-7:** Percentage of subclinical mastitis based on CMT result.

Sample	Total no.	Positive (%)	Trace (%)	Weak (%)	Distinct (%)	Strong (%)
Milk of Cow	20	16 (80%)	5 (31.25%)	2 (12.5%)	3 (18.75%)	6 (37.5%)

CMT=California mastitis test

### Isolation and characterization of *S. aureus*

Results showed that *S. aureus* ferments menthol sugar, changing its color from red to yellow and showing aerobic growth on mannitol agar medium. These isolates were examined under a light microscope after Gram-staining. Cells were Gram-positive cocci (in grape-like irregular clusters); after further purification by biochemical testing, cells were catalase positive but showed negative slide coagulase, coagulase test, and oxidase results. Thirty (83.33%) of suspected *S. aureus* samples were isolated using standard microbiological methods, with statistically significant results (p > 0.05) (Table- 8).

**Table-8:** Result of *S. aureus* isolated from all samples.

Samples	Total no.	*S. aureus* isolates by con. micro. tech no. %
Subclinical mastitis	16	14 (87.5)
Raw milk from local markets	20	16 (80)
Total	36	30 (83.33)

*S. aureus=Staphylococcus aureus*

### Molecular techniques

#### DNA extraction

**Figure-1:**
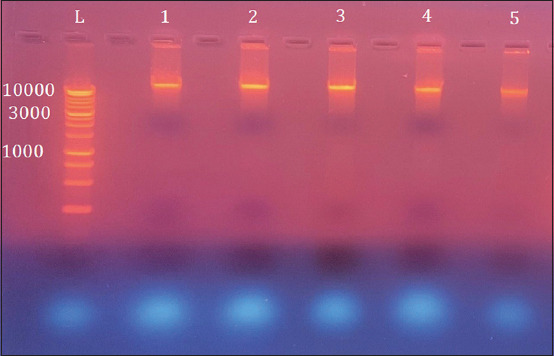
Electrophoresis of extracted DNAs in 1% agarose gel stained with ethidium bromide at 100 volt and 80 am for 1 h. Lane (L): Ladder marker (10000–100 bp). Lanes (1–5): Whole DNAs of positive isolated samples by culture.

All bacterial genomes of positive cultures were isolated and subsequently electrophoresed on 1% agarose gel (Figure-1).

#### Specific 16S rRNA region amplification for LAB

**Figure-2:**
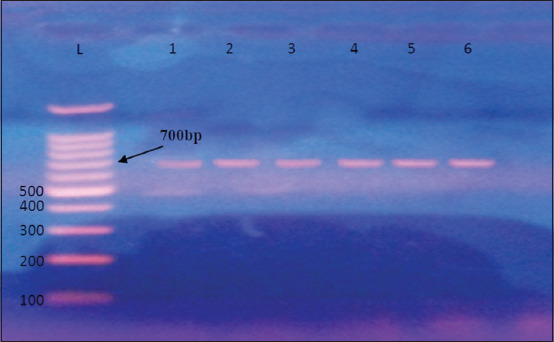
Electrophoresis of amplified polymerase chain reaction products of cow and sheep white soft local cheese lactic acid bacteria isolates targeting *16S rRNA* gene in 2% agarose gel stained with ethidium bromide at 100 volt and 80 am for 1 h. Lane (L): Ladder marker (1000–100 bp). Lanes (1–6): Positive polymerase chain reaction products at 700 bp.

After DNA isolation, PCR was used to amplify the 16S rRNA region. Polymerase chain reaction results were seen using agarose gel electrophoresis with UV light illumination. The amplification products were 700 bp long (Figure-2). The 16S rRNA molecular detection of LAB showed that 28 of 35 (80%) isolates from cow white soft local cheese samples were positive for this gene, whereas 18 of 32 (56.25%) isolates from sheep white soft local cheese samples were significantly positive (p < 0.05; Table-9).

**Table-9:** Distribution of LAB in white soft local cheese sources according to PCR results

Type of samples	No. of LAB isolates	16S rDNA gene	

		Positive	Negative
Cow	35	28 (80%)	7 (20%)
Sheep	32	18 (56.25%)	14 (43.75%)
Total	67	46 (68.65%)	21 (31.34%)

PCR=Polymerase chain reaction, LAB=Lactic acid bacteria

#### Ability of S. aureus to produce virulence factors (mecA detection)

**Figure-3:**
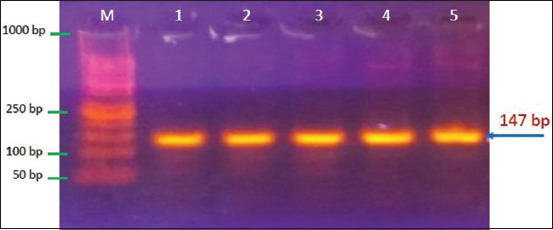
Electrophoresis of amplified polymerase chain reaction products of *Staphylococcus aureus* isolates targeting *mecA* gene in 1.5% agarose gel stained with ethidium bromide at 100 volt and 80 am for 1 h. Lane (L): Ladder marker (1000–100 bp). Lanes (1–6): Positive polymerase chain reaction products at 147 bp.

PCR was used to identify *S. aureus* isolates carrying the *mecA*. Results showed that some MRSA isolates harbor the *mecA*, with a molecular weight of 147 bp (Figure-3). This gene was positive in 14 of 16 (87.5%) raw milk samples from local markets (p < 0.05; Table-10).

**Table-10:** Detection of MRSA by genotypic methods.

Type of samples	No. of isolates	*mecA gene*	

		Positive	Negative
Subclinical mastitis	14	12 (85.71%)	2 (14.28%)
Raw milk from local markets	16	14 (87.5%)	2 (12.5%)
Total	30	26 (86.66%)	4 (13.33%)

MRSA=Methicillin-resistant *Staphylococcus aureus*

#### Antibiotic sensitivity for MRSA

**Figure-4:**
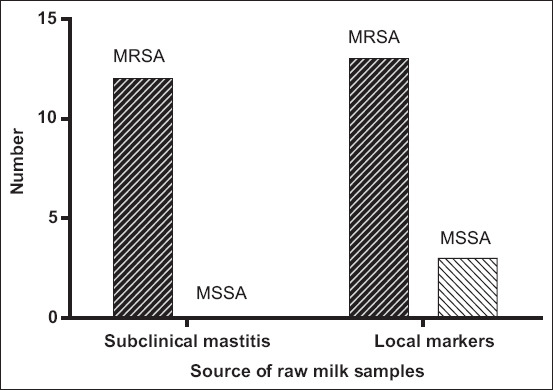
Methicillin drug susceptibility test.

The methicillin resistance of all *S. aureus* isolates was investigated using the methicillin drug susceptibility test. Results showed subclinical mastitis from all 12 isolates (100%) showed resistance to methicillin (MRSA), whereas 13 isolates (81.25%) from raw milk from local markets were resistant to methicillin and 3 isolates (18.75%) were sensitive to methicillin (methicillin-susceptible *Staphylococcus aureus* [MSSA]) (Figure-4).

#### Biofilm formation

**Figure-5:**
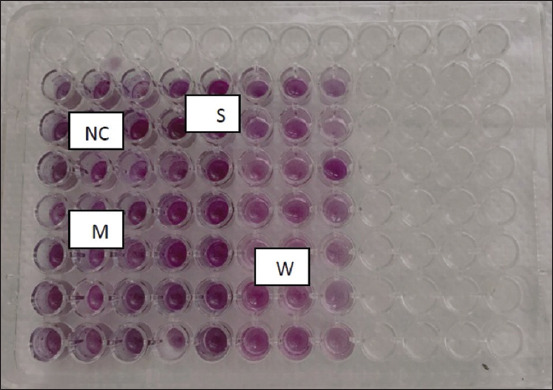
The biofilm formation by *Staphylococcus aureus* on the microtiter plate NC=Negative control, S=Strong, M=Moderate, W=Weak.

Different levels of biofilm development were found as strong, weak, and moderate (58.33%, 25%, and 16.66%, respectively) using the microtiter plate assay that tested all isolates of *S. aureus* (Table-11 and Figure-5). In MRSA and MSSA, various levels of biofilm formation were found to be none, mild, and moderate. Among MRSA isolates, 88% of isolates produced biofilm, whereas 12% did not. In addition, whereas 100% of MSSA isolates could create biofilm, only 27.27% of biofilm producer isolates were weak, and only 72.72% were moderate producers. Furthermore, 66.66% of biofilm producer isolates were moderate, and only 33.33% were weak. There were no obvious differences between MRSA and MSSA regarding biofilm production (p < 0.05).

**Table-11:** Results of standard conditions microtiter plate assay for *S. aureus* biofilm formation.

Samples	Total no. of isolates	Methicillin-resistant and sensitive isolates	Total no. of biofilm	Biofilm production		

				No. of strong (%)	No. of weak (%)	No. of moderate (%)
Cow milk	12	MRSA	12	7 (58.33%)	3 (25%)	2 (16.66%)
		MSSA	0	0	0	0
Cow milk of market	16	MRSA	9	4 (44.44%)	5 (55.55%)	0
		MSSA	5	0	3 (60%)	2 (40%)

*S. aureus=Staphylococcus aureus*, MRSA=Methicillin-resistant *Staphylococcus aureus*, MSSA=Methicillin-susceptible *Staphylococcus aureus*

#### Effect of crude bacteriocin on biofilm of S. aureus

**Figure-6:**
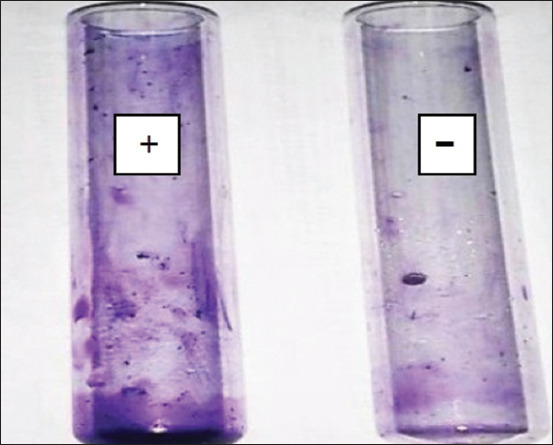
Inhibitory activity of crude bacteriocin against the biofilm of *Staphylococcus aureus*. (-): No adherent cells appeared on the walls of test tubes after treatment with crude bacteriocin. (+): The appearance of adherent cells on the walls of the test tubes.

The effect of crude bacteriocin from LAB on biofilm formation inhibition by MRSA pathogenic bacteria was investigated before and after treatment of MRSA with crude bacteriocin for 24 h. The inhibitory effect was clear against MRSA. Results showed a clear effect of bacteriocin on MRSA isolates that can attach to tube walls after crystal violet staining compared to MRSA-containing control tubes without bacteriocin (Figure-6).

## Discussion

In wealthy nations, LAB has a lengthy history of modern applications. Over the past two decades, other nations have been compelled to make serious efforts to isolate, identify, and improve local LAB for use in the industry due to the significance of these bacteria for the industry and health improvement [29]. A habitat that supports a complex and diversified microbial community is demonstrated by white soft local cheese [30].

Most studies indicated that the culture medium in use lacks sufficient selectivity [31]. Accordingly, genomic DNA from isolates was extracted using the procedure recommended by the manufacturer of the DNA kit, and extracted DNA was visible using agarose gel electrophoresis under UV light. Strains isolated from cow and sheep white soft local cheese using MRS medium were regarded as LAB because they could survive in anaerobic conditions and were Gram-positive and catalase-negative [32].

### Polymerase chain reaction identification of LAB and MRSA

Many studies highlighted the lack of adequate selectivity in the employed culture medium, even for LAB [33]. Accordingly, genomic DNA from isolates was extracted using the procedure recommended by the manufacturer of the DNA kit, and extracted DNA was visible using agarose gel electrophoresis with UV light illumination. All 67 isolates of cows and sheep that showed Gram-positive results but negative-catalase reactivity were then submitted to 16S rRNA-based PCR identification. Lactic acid bacteria were used to identify the isolates (64 isolates in total; 28 cows, 18 sheep, and 18 white soft local cheese). The employment of 16S rRNA at 700 bp in identifying white soft local cheese LAB isolates by PCR was in agreement with others [34], who found that the V1 region (90 bp) of the *16S rRNA* gene was enough to identify isolates correctly and reliably, with mutations that allowed the separation of their species and subspecies. In this study, 67 LAB isolates were obtained using conventional phenotypic techniques. However, when these isolates were sequentially subjected to 16S rRNA-based PCR, 46 positive 16S rRNA carrier LAB were obtained. This result was confirmed as molecular methods are important for bacterial identification and may be more accurate for LAB than conventional phenotypic techniques [35]. In accordance with these data, isolates from raw milk carrying the *16S rRNA* gene were recognized as LAB, as typically seen in studies that look into their presence in native microbiota from food systems [36]. The prevalence of staphylococcal mastitis in dairy animals varies greatly between nations and may result from various infection management strategies. Comparing this study to those published by others is challenging because the prevalence of *S. aureus* as a mastitis-causing agent varies depending on the region, how the animals are handled, and the sanitary conditions present during milking [37]. Subclinical mastitis is a serious issue because it has no outward signs [38]. In agreement with several studies, current CMT findings showed that 16 (80%) milk samples were positive. In a study conducted in Iraq, the prevalence of subclinical mastitis among the cows in Al Sulaymaniyah Governorate was 38.89% [39], whereas 52% of the cows in Diyala Province had subclinical mastitis [40]. Compared to another study, more cows (68.44%) had subclinical mastitis, which was identified by CMT in Basrah Province [41].

### Ability of *S. aureus* to produce virulence factors (mecA detection)

Examination of the *mecA* is one of the most effective approaches to MRSA isolation [42]. The lowest β-lactam potential alters protein (PBP2a) is encoded by the *mecA*, found on SCCmec-resistant genomes [43]. In this study, the rate of MRSA according to the *mecA* detected by PCR was 86.66%, which is consistent with findings from regional studies examining mastitis milk from dairy animals [44]. In addition, Hammadi and Yousif [45] reported that 88% of *S. aureus* cases were MRSA, Al-Jebouri and Mdish [46] found only 10% of *S. aureus* cases to be MRSA, and Hammadi and Yousif [45] reported higher results. This outcome was consistent with numerous studies demonstrating that every MRSA isolates contained *mecA* [47]. Other studies have suggested that methicillin resistance can occur in the absence of *mecA* and that MRSA may possess additional resistance mechanisms, such as changed target sites or possibly decreased drug accumulation. The *mecA* absence could result from a detection error [48].

### Methicillin-resistant *S. aureus* antibiotic sensitivity

The potential of MRSA strains to develop resistance to antimicrobial treatment makes them cause major nosocomial diseases and spread worldwide in recent years [49]. Thus, prompt identification of these infections and detection of methicillin resistance are essential for promoting successful treatment and preventing the spread of illness [50]. The rate of MRSA identified in this study using the methicillin disk diffusion approach was 100%. The rate of methicillin resistance in *S. aureus* isolated from mastitis milk of dairy animals was 61%, consistent with findings of local research (60%) [51]. In addition, Idbeis [52] reported that 55% of people were resistant to methicillin, whereas better outcomes were observed by Akil and Muhlebach [53]. Kadiyala *et al*. [54] reported that only 10% of *S. aureus* cases were MRSA. Methicillin-resistant *S. aureus* strains were distinguishable from methicillin-susceptible strains using such tests [55].

### Biofilm formation using standard conditions

Results of biofilm formation in *S. aureus* isolates agreed with several studies that established the capacity of *S. aureus* isolates to form biofilm [56]. There were no significant differences (p > 0.05) in the percentage of weak and moderate biofilm formation between MRSA and MSSA isolates, in agreement with earlier studies [57] that demonstrated the ability of MRSA to spread on hard surfaces and subsequently result in biofilm formation [52].

### Effect of crude bacteriocin on *S. aureus* biofilm

Bacteriocin had a clear effect on MRSA isolates (Figure-6). Bacteriocin from LAB was deemed effective in inhibiting the biofilm formation of *S. aureus* and other Gram-negative bacteria [58]. These results did not agree with Nawaz *et al*. [59], who showed that bacteriocin has little efficacy against MRSA, affecting only 1 of 50 strains. Other studies have confirmed that bacteriocin, especially nisin, is more effective than antibiotics and used to prevent and treat infections caused by MRSA associated with biofilm composition [60].

## Conclusion

LAB demonstrates a high ability to produce bacteriocin. Crude bacteriocin from LAB has a restrictive effect on biofilms produced by MRSA; thus, it can be used to reduce the pathogenicity of this bacterium. Further studies are necessary to detect the protective role of bacteriocin on severe MRSA infections or other invasive bacterial infections.

## Authors’ Contributions

HKI: Designed and supervised the study, collection of samples, and isolation of S. aureus. KSM: Detection of MRSA and Biofilm Formation Assay. GKB: Testing the effect of crude bacteriocin on biofilm formation of S. aureus. HAJG: Lead the molecular examination and statistical analy­sis and drafted and revised the manuscript. All authors participated in molecular examination and statistical analysis. All authors have read, reviewed, and approved the final manuscript.
